# Comparative genomic analysis of Clostridioides difficile strains in Mexico: insights into virulence and resistance

**DOI:** 10.1099/acmi.0.001132.v3

**Published:** 2026-07-29

**Authors:** C. Ortiz-Flores, A. Romero-Rodríguez, R. Villanueva-Enríquez, Corina-Diana Ceapă, N. Gómez-Rivera, M. Velázquez-Romero, F. Mendoza-Torres, M.M.P Arenas-Hernández, C. Martínez de la Peña

**Affiliations:** 1 Posgrado en Microbiología, Centro de investigaciones en ciencias Microbiológicas, Instituto de Ciencias, Benemérita Universidad Autónoma de Puebla, Ciudad Universitaria, Puebla, Puebla, 72592, Mexico; 2 Departamento de Biología Molecular y Biotecnología, Instituto de Investigaciones Biomédicas, Universidad Nacional Autónoma de México, Mexico City, 04510, Mexico; 3 Microbiology Laboratory, Department of Natural Products Chemistry, Institute of Chemistry, Universidad Nacional Autónoma de México, Mexico City, 04510, Mexico; 4 Licenciatura en Biomedicina, Facultad de Medicina, Centro de Investigaciones en Ciencias microbiológicas, Instituto de Ciencias, Benemérita Universidad Autónoma de Puebla, Ciudad Universitaria, Puebla, Puebla, 72592, Mexico; 5 Laboratorio de Biología Celular y Productos naturales, Escuela Nacional de Medicina y Homeopatía, Instituto Politécnico Nacional, México City, 07320, Mexico

**Keywords:** antimicrobial resistance, CDI, clades, *Clostridioides difficile*, comparative genomics, genomic surveillance, hypervirulent lineages, Mexico, whole-genome sequencing (WGS)

## Abstract

*Clostridioides difficile* infection (CDI) remains a major global health threat due to the emergence of hypervirulent, multidrug-resistant lineages. However, the evolutionary dynamics and resistance-associated genomic profiles of strains circulating in underrepresented regions, such as Mexico, remain poorly characterized. Here, we present a comprehensive genomic and phylogenetic analysis of 77 Mexican *C. difficile* strains compared with 74 strains from other parts of the world. Using whole-genome sequencing and core-genome MLST, we identified 19 sequence types (STs) grouped across 3 clades, with hypervirulent ST01 dominating clade 2. Virulome analysis showed conserved toxin gene profiles (*tcdA*, *tcdB* and *cdtAB*) across strains, while clade-specific differences were observed in adhesion and survival genes. These variations, particularly pronounced in clade 2 strains from both global and Mexican collections, may contribute to enhanced persistence and transmissibility. Pangenome analysis of 151 genomes highlighted distinct genomic architectures. Clade 2, enriched in ST01 epidemic lineages, contained 5,480 genes (58% core, 42% accessory), showing a compact structure consistent with recent clonal expansion. In contrast, clade 1 displayed the highest diversity, with 8,584 genes (30% core, 70% accessory), indicative of an open and dynamic pangenome, while clade 4 showed a smaller, more conserved profile (4,784 genes, 63% core). These findings underscore the contrasting evolutionary strategies among clades. Notably, Mexican ST01 strains exhibited a distinct resistome, including the high prevalence of the *vanG* operon and the VanR T115A substitution (94% vs. 23% globally), as well as near-complete prevalence of the P*nimB^G^* mutation associated with reduced metronidazole susceptibility. This pattern may reflect local selective pressures associated with antimicrobial exposure. Phenotypic susceptibility testing of newly sequenced isolates showed that most ST01 strains remained susceptible to metronidazole and vancomycin despite carrying resistance-associated determinants. Our findings highlight the urgent need to recognize hypervirulent and resistant *C. difficile* lineages arising outside traditional surveillance regions. These Mexican strains not only reflect regional antibiotic usage patterns but also represent a potential reservoir of globally significant resistance traits. This work underscores the importance of integrating genomic surveillance across all continents to refine treatment protocols, prevent outbreaks and contain the spread of resistant CDI.

**Impact Statement**

This study demonstrates that *Clostridioides difficile* strains circulating in Mexico, particularly ST1/RT027-associated lineages, harbour distinct resistance-associated determinants and phylogenomic characteristics within the Latin American context. Combined genomic and phenotypic analyses highlight the regional circulation of multidrug-resistant lineages and emphasize the need for globally inclusive genomic surveillance of emerging hypervirulent *C. difficile* strains.

## Data Summary

The raw sequencing reads are available from the National Center for Biotechnology Information under the BioProject accession number PRJNA1125493 (Supplementary Table).

## Introduction

*Clostridioides difficile* infection (CDI) is one of the leading causes of healthcare-associated diarrhoea worldwide, contributing significantly to patient morbidity, prolonged hospitalizations and increased healthcare costs [1, 2]. Over the last two decades, the emergence of hypervirulent and multidrug-resistant strains, particularly those belonging to clade 2 such as sequence type 01 (ST01, ribotype 027), has dramatically reshaped the epidemiology of CDI, leading to more severe infections and higher mortality rates [3, 4].

While intensive genomic surveillance has elucidated the evolutionary dynamics of *C. difficile* in North America and Europe, Latin America remains critically underrepresented in global studies [5]. This lack of regional data constitutes a significant blind spot in understanding how local antibiotic usage, healthcare infrastructure and epidemiological pressures may influence the evolution of RT027-associated and drug-resistant *C. difficile* lineages. Early and accurate identification of *C. difficile* is essential for epidemiological studies and infection control.

Whole-genome sequencing (WGS) has provided valuable insights into the genomic diversity, evolution and antimicrobial resistance of *C. difficile* [4, 6, 7]. In Mexico, there is only one report that shows a high prevalence of hypervirulent strains, with ST01/RT027 representing between 30 and 40% of clinical isolates [8]. Nevertheless, comparative genomics studies remain limited. Molecular typing techniques, such as MLST and core-genome MLST (cgMLST), have been essential in defining the phylogenetic structure of *C. difficile*, classifying it into five main clades (C1–C5) with distinct epidemiological and genomic characteristics. The MLST scheme is based on seven housekeeping genes, including *tpi* (triosephosphate isomerase), which encodes an essential glycolytic enzyme. Owing to its conservation across all *C. difficile* lineages, *tpi* is commonly used as a species-specific marker in molecular assays and plays a central role in phylogenetic and epidemiological studies [9]. Among them, clade 2, which includes the hypervirulent RT027/ST01 strain, has been strongly associated with increased virulence and antimicrobial resistance [10].

The pathogenicity of *C. difficile* is primarily mediated by toxins A (*tcdA* gene) and B (*tcdB* gene), encoded in the pathogenicity locus (*PaLoc*). TcdA disrupts intestinal epithelial integrity, increasing permeability and triggering inflammation, whereas TcdB is a more potent cytotoxin that alters Rho GTPases, leading to cytoskeletal disruption and cell apoptosis [11]. Epidemic RT027/ST01-associated strains frequently carry characteristic *tcdC* polymorphisms, including truncating deletions that have been proposed to influence toxin regulation [12].

In recent years, an increasing diversity of TcdA and TcdB toxin subtypes has been identified, reflecting differential evolution among *C. difficile* clades [13]. TcdA exhibits variations in its receptor-binding domain, modulating its cytotoxicity. In contrast, TcdB shows greater functional diversity, with at least five subtypes (TcdB1–TcdB5), each differing in toxicity and interaction with human epithelial cells [14]. Its genetic variability plays a crucial role in disease severity and the spread of hypervirulent lineages, underscoring its importance in strain subtyping [13].

In addition to the major toxins TcdA and TcdB, certain *C. difficile* lineages harbour the binary toxin locus (*cdtA* and *cdtB*), collectively known as CDT (*C. difficile* binary toxin or *C. difficile* transferase), together with the regulatory gene *cdtR*. The binary toxin has been associated with enhanced adherence to intestinal epithelial cells, microtubule-based protrusion formation and increased bacterial colonization [15, 16]. The distribution of the CDT locus is strongly clade-dependent and is particularly enriched in epidemic clade 2 RT027/ST01-associated strains, whereas it is absent or less frequent in other phylogenetic clades. Recent studies have also shown that *cdtR*, a positive regulator of CDT production, may influence toxin expression in RT027 strains, although its functional role outside these epidemic lineages remains incompletely understood [17].

The standard treatment for CDI includes antibiotics such as vancomycin, metronidazole and fidaxomicin [18]. A key genetic mechanism of interest is the *vanG* cluster, which encodes a peptidoglycan modification pathway that reduces vancomycin affinity, potentially decreasing its efficacy [19]. However, the presence of *vanG* in *C. difficile* does not always confer this phenotypic resistance, as its expression may be low or regulated by cellular factors [20]. Notably, mutations in the transcriptional regulator *vanR*, such as *vanR* T115A, have been associated with increased *vanG* expression, suggesting a possible correlation with clinical vancomycin resistance in specific *C. difficile* lineages [21]. A key resistance determinant in *C. difficile* is the *nimB* gene, which encodes a 5-nitroimidazole reductase implicated in metronidazole resistance. The P*nimB^G^* mutation, located in its promoter, has been linked to increased gene expression, reducing antibiotic activity within the bacterial cell [22]. In addition to chromosomal resistance mechanisms, plasmid-mediated metronidazole resistance has been associated with the transferable plasmid pCD-METRO, which carries genes linked to reduced susceptibility and has been identified predominantly in epidemic *C. difficile* lineages [23, 24].

Recent research has demonstrated that localized antibiotic selection pressures can shape the resistome and virulome of bacterial pathogens, promoting the emergence of regionally adapted strains with distinct genetic profiles [23, 25]. In Mexico, where metronidazole and vancomycin remain frontline therapies for CDI and other diseases, and where antimicrobial stewardship programmes are still evolving, selective pressures may favour the persistence and spread of resistant *C. difficile* lineages. However, the genomic characteristics, phylogenetic structure and antimicrobial resistance mechanisms of Mexican *C. difficile* strains have not been comprehensively investigated. Addressing this gap is crucial not only for improving local infection control strategies but also for anticipating the emergence of globally significant resistance traits.

In the present study, we performed WGS of 11 strains and cgMLST of 77 *C*. *difficile* strains isolated in Mexico, comparing them with 74 genomes from other regions of the world. Our goal was to characterize the genomic diversity, virulence factors and resistance determinants of Mexican strains; evaluate their evolutionary relationships; and place them within a broader global epidemiological framework. By doing so, we aim to illuminate the hidden dynamics shaping the global spread of *C. difficile* and underscore the importance of including underrepresented regions in surveillance efforts.

## Methods

### Isolation and identification of *C. difficile*

WGS was performed on 11 *C*. *difficile* strains isolated in our research lab from stool samples obtained from hospitalized patients diagnosed with CDI without information about their treatment or response to it. All metadata associated with these isolates are provided in Table S2 (available in the online Supplementary Material). *C. difficile* strains were selected among the first isolates obtained in the research lab since routine isolation is not routinely performed. Stool samples were donated from three hospitals in Puebla and a paediatric hospital in Mexico City between 2018 and 2023. The samples were collected after the respective clinical laboratories completed necessary diagnostic tests and had completed routine diagnostic processing, eliminating the need for informed patient consent. For *C. difficile* isolation and identification, stool samples were treated with 96% ethanol at room temperature for 50 min, followed by centrifugation at 4,000 r.p.m. for 10 min. The resulting cell pellets were inoculated onto Cycloserine Cefoxitin Fructose agar (CCFA-T) supplemented with 0.1% taurocholate and incubated anaerobically at 37 °C for 48 h using an anaerobic jar with the AnaeroCult system (Millipore, Sigma-Aldrich). *C. difficile* isolates were identified based on colony morphology, Gram staining, amplification of the *tpi* gene [26] and specific 16S rRNA gene sequencing. All isolates were stored at −80 °C in brain heart infusion broth supplemented with 50% glycerol for further analysis. The *C. difficile* R20291 strain was used as a positive control for different studies [27].

### Genomic DNA extraction

Genomic DNA was extracted and purified using the phenol-chloroform method, following the protocol described by Andreou [28]. DNA samples were stored at −20 °C for subsequent use and sequencing.

### WGS and bioinformatics analysis

Genomic sequencing was conducted using the Illumina HiSeq 2500 platform. Eight strains were sequenced in collaboration with the Institute of Chemistry at Universidad Nacional Autónoma de México (UNAM), in partnership with MGI, Applikon and Illumina, at no cost to the research group, while three strains were sequenced at SeqCenter in Pittsburgh, USA.

Genomes were assembled *de novo* using SPAdes v3.13.0 [29] on the BV-BRC v3.31.12 (Bacterial and Viral Bioinformatics Resource Center) online platform. Taxonomic identification was conducted using default parameters. Assembly quality was assessed using QUAST v5.0.0 [30] on the Galaxy platform (https://usegalaxy.eu/), and genomes with contamination or low coverage (<33×) were excluded. Contigs were annotated using the Prokka v1.13 pipeline [31] in Galaxy. The 11 genomic sequences were deposited in NCBI under BioProject accession number PRJNA1125493, with individual accession numbers detailed in Table S1.

### MLST and clade assignment

MLST and clade assignment for all isolates were performed using the *C. difficile* MLST scheme available in the PubMLST database (http://pubmlst.org/cdifficile), which employs seven housekeeping genes (*adk*, *atpA*, *dxr*, *glyA*, *recA*, *sodA* and *tpi*), following the methodology described by Griffiths *et al.* [32] (Table S6).

### Genome collection and selection for comparative analysis

The study included our 11 newly sequenced Mexican strains along with 66 previously reported *C. difficile* strains isolated from humans in Mexico from studies by Aguilar-Zamora *et al*. [8], Zhao *et al*. [33] and Guerrero-Araya [5]. The 66 Mexican *C. difficile* genomes were collected through searches in public databases, including NCBI [Sequence Read Archive (SRA) and GenBank], using the terms ‘*Clostridioides difficile*’ AND ‘Mexico’, along with a manual review of published studies. Genomes from Aguilar-Zamora *et al*. [8], Zhao *et al*. [33] and Guerrero-Araya *et al*. [5], as well as genomes generated in the present study, were included. After removing duplicates and reviewing metadata, genomes were selected based on the availability of paired-end reads, acceptable assembly quality metrics and verifiable geographic origin, resulting in 66 Mexican genomes retrieved from public databases. Metadata, including year of isolation, geographic origin, sequence type (ST), ribotype and source of isolation, are provided in Table S1. Ribotype inference was performed based on the MLST–ribotype correspondence framework described by Zhao *et al*. [33]. These sequences were retrieved from NCBI and the China National GeneBank DataBase (CNGBdb) (Table S1). Additionally, 74 complete genomes from other countries were compiled from studies by He *et al*. [3], Zhao *et al*. [33], Pu *et al*. [34], Boekhoud *et al*. [23], Guerrero-Araya *et al*. [5], Muñoz *et al*. [35], Pecora *et al*. [36], Stabler *et al*. [27] and Gu *et al*. [37], sourced from public databases using the same parameters but prioritizing representation of the same major clades and epidemic lineages identified among Mexican isolates (NCBI, EnteroBase and CNGBdb) (Table S1). All retrieved genomes were assembled from paired-end Illumina reads using the BV-BRC platform assembly service, which enabled direct upload of SRA files via NCBI accession numbers. Quality control and assembly followed the same pipeline as described above.

### Phylogenetic analysis based on cgMLST

Maximum likelihood phylogenetic trees were constructed based on cgMLST to assess strain relationships and evolutionary divergence. The analysis was performed using the cgMLSTFinder v1.2 tool [38, 39] available on the Center for Genomic Epidemiology platform (https://cge.food.dtu.dk/services/cgMLSTFinder). Minimum spanning trees (MSTs) were subsequently generated to visualize cgST distribution among examined strains using the PHYLOViZ Online platform [40] (https://online.phyloviz.net/index) project name *C. difficile* Mexico 2025.

### Pangenome analysis and phylogenetic relationships

Pangenome analysis of the 151 *C*. *difficile* genomes was performed using Roary v3.11.2 [41] on the Galaxy platform. Roary enabled alignment of core genomes based on genome annotation files, generating a matrix of core and accessory gene presence/absence. The pangenome analysis results were integrated with phylogenetic trees generated from cgMLST and visualized using Phandango v1.3.0 [42].

### Identification of virulence factors and antimicrobial resistance-associated genes

Virulence gene identification was conducted using ABRicate v1.0.1 [43] with the Virulence Factor Database (VFDB) [44] and AMRFinderPlus v3.10.24 [45]. TcdB toxin subtyping was performed using the publicly available DiffBase database [14] (https://diffbase.uwaterloo.ca/). Antimicrobial resistance gene identification and detection of point mutations were performed using AMRFinderPlus v3.10.24 from the NCBI database and the RGI 6.0.2 tool (Resistance Gene Identifier) within the Comprehensive Antibiotic Resistance Database (CARD 3.2.7) [46]. Comparative gene presence/absence analyses were visualized using Microreact (https://microreact.org/) [47]. Detection of pCD-METRO was performed by BLASTn against the reference sequence of pCD-METRO (GenBank: OM972905.1) with coverage >80%.

To complement the automated identification of virulence determinants, additional comparative genomic analyses were performed for loci showing incomplete or inconsistent detection by standard bioinformatic pipelines. Detection of *slpA* was performed in three progressive stages. Initially, genomes were screened using ABRicate v1.0.1 against the VFDB database applying thresholds of ≥90% nucleotide identity and ≥80% coverage. Genomes negative by this approach were subsequently reanalysed by BLASTn using the *slpA* allele from strain 630 (WP_011861686; 2,160 bp) as reference, applying thresholds of ≥70% identity, ≥20% coverage and e-value ≤1×10⁻⁵. Genomes remaining negative were finally subjected to a multi-allelic BLASTn search using 61 *slpA* reference sequences retrieved from NCBI (May 2026) with thresholds of ≥50% identity, ≥50% coverage and e-value ≤1×10⁻⁵. Complementary tblastn searches were additionally performed using the SlpA protein sequence from strain 630 (WP_011861686; 719 aa; e-value ≤1×10⁻⁵).

### Analysis of CDT locus integrity

To evaluate the integrity of the CDT locus in clade 1 strains carrying *cdtR* in the absence of detectable *cdtA/cdtB* genes, additional comparative analyses were performed using the CDT locus from reference strain R20291 (FN545816.1). BLASTn searches were used to assess sequence conservation and coverage across *cdtR*, *cdtA* and *cdtB*. Predicted *cdtR* coding sequences were translated and aligned using MAFFT v7.525 to verify structural conservation of the regulator. Genomic synteny of the CDT locus was additionally evaluated by comparison against the reference locus. Fragmented coverage patterns affecting *cdtA* and *cdtB* together with conservation of intact *cdtR* sequences were interpreted as evidence consistent with pseudogenization of the structural CDT genes.

### Antimicrobial susceptibility testing

Antimicrobial susceptibility testing for metronidazole, vancomycin and clindamycin was additionally performed for the 11 newly sequenced isolates generated in this study using the agar dilution method according to CLSI guidelines and interpreted using CLSI breakpoints. The MIC range evaluated did not include concentrations below 4 µg ml^−1^; therefore, retrospective interpretation using EUCAST criteria was not possible. Complete phenotypic susceptibility data are provided in Table S8.

## Results

### Identification of *C. difficile* strains

Presumptive identification of *C. difficile* isolates was performed by PCR of *tpi* gene, and the amplification of the four toxin genes were performed. The presence of *C. difficile tpi* gene was confirmed in all 11 analysed strains, which were subsequently subjected to WGS. Toxin gene detection by PCR revealed that two strains (18.2%) did not carry any of the evaluated toxin genes (*tcdA*, *tcdB*, *cdtA* and *cdtB*). In contrast, 9 out of 11 strains (81.8%) were positive for *tcdA* and *tcdB*, which encode the primary toxins responsible for *C. difficile* pathogenicity. The MX_CD799 strain was the only strain (9.1%) that also carried *cdtA* and *cdtB*, responsible for producing binary toxin CDT (Table S3).

### Phylogenetic analysis based on cgMLST

**Fig. 1.**
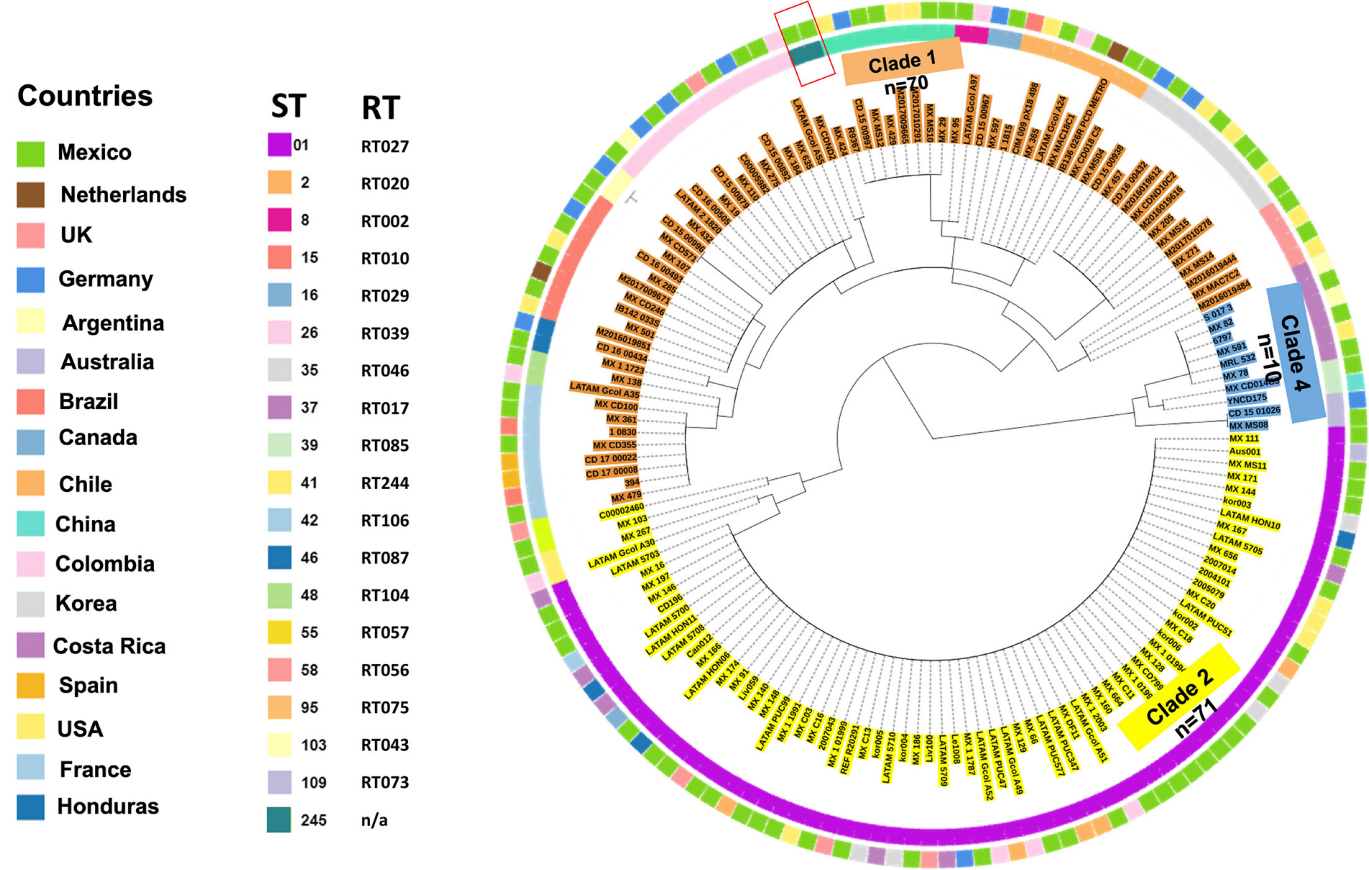
Phylogenetic analysis based on cgMLST of Mexican and global strains. Circular neighbour-joining phylogenetic tree based on cgMLST of 151 *C*. *difficile* strains, showing the phylogenetic relationships among 19 STs from Mexican strains and global strains. The concentric circles, organized progressively from the innermost, are detailed as follows: Circle 1 represents the phylogenetic tree grouped into three clades, indicated by different colours: orange for clade 1, yellow for clade 2 and blue for clade 4. Circle 2 shows the distribution of STs among the strains. Circle 3 indicates the countries of origin of the analysed strains. Red box indicates ST245. Phylogenetic tree was constructed using cgMLSTFinder v1.2 and edited and visualized with iTOL v6.

To analyse the genetic relationships and evolutionary divergence of 77 Mexican strains together with 74 global strains, maximum-likelihood phylogenetic trees based on cgMLST profiles were constructed. This analysis classified the strains into 19 STs, distributed across 3 main clades: clade 1 (C1) with 70 strains (46.36%), clade 2 (C2) with 71 strains (47.02%) and clade 4 (C4) with 10 strains (6.62%) (Fig. 1 and Table S2). ST01 was the most dominant ST for clade 2, accounting for 44% (*n*=34) of the 77 Mexican strains. However, one ST (ST-245) was exclusively identified in Mexican strains and was absent in public databases (red box, Fig. 1).

**Fig. 2.**
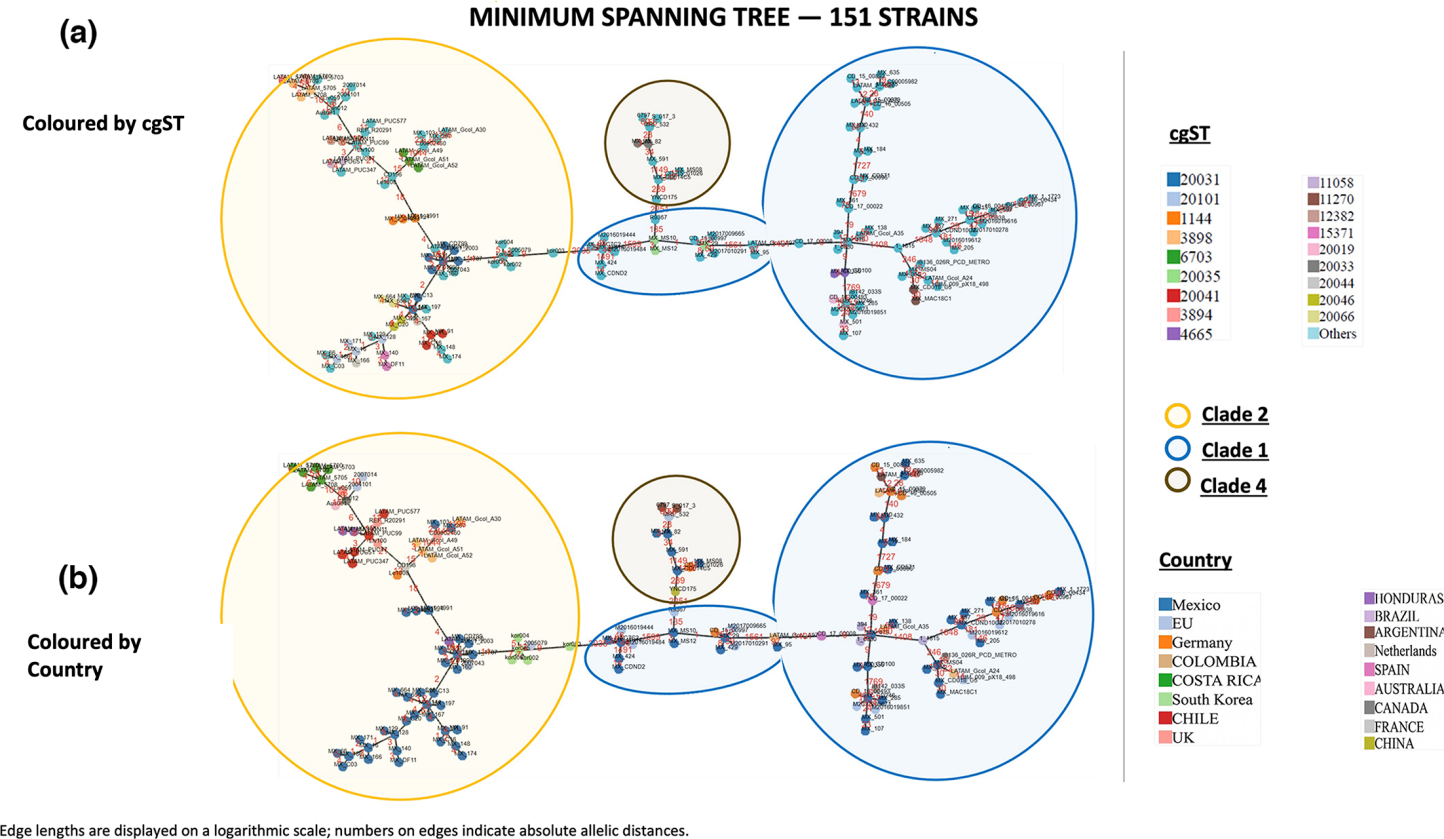
MSTs based on cgMLST analysis of Mexican and global strains. The trees are divided into the three identified clades, with each node representing a specific strain. (a) Coloured nodes indicate cgST allele profiles that are repeated at least once, while grey nodes represent unique cgST profiles that do not appear in other strains. (b) Coloured nodes indicate strain origin country. Phylogenetic trees were constructed and edited using PHYLOViZ Online.

MSTs based on cgMLST analysis revealed a clear clade-based clustering of the analysed strains (Fig. 2a). Seven strains (5%) shared cgST20031, primarily among ST01 strains from clade 2 in Mexican isolates, indicating lower genetic variability within this lineage. Examination of the epidemiological metadata showed that three isolates from Guadalajara, Mexico, were recovered in the same year, suggesting close genomic relatedness and possible local persistence of a circulating lineage. In contrast, five isolates from Mexico City sharing the same cgST were isolated over a broader time span (2014–2019), arguing against a single outbreak and instead suggesting persistence and circulation of a stable lineage in this region. As illustrated in Fig. 2(b), repeated cgSTs were predominantly observed among Mexican clade 2 strains (denoted in green), whereas strains from other clades exhibited greater genetic diversity despite sharing the same ST.

**Fig. 3.**
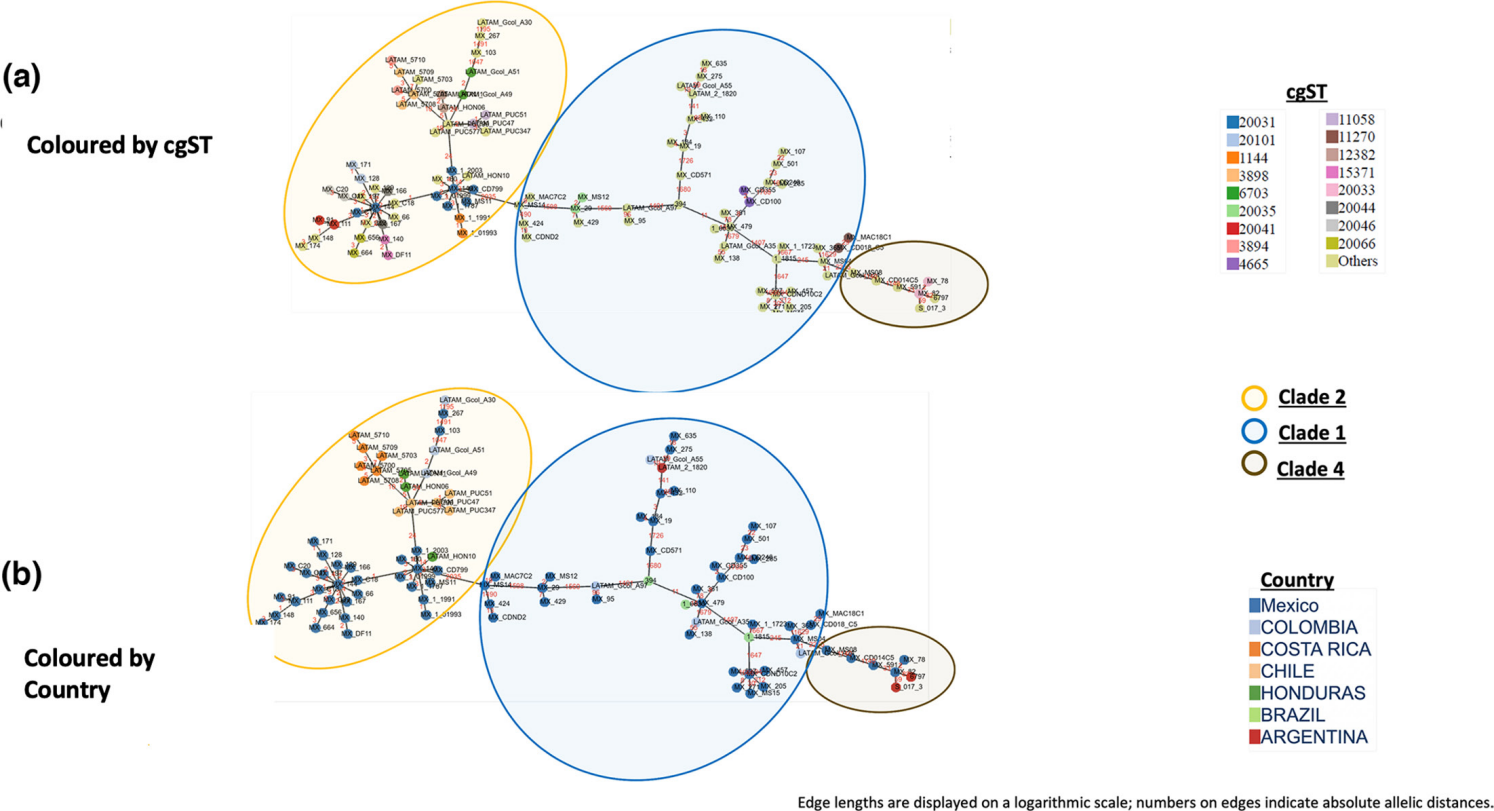
MST based on cgMLST profiles of Latin American *C. difficile* isolates included within the global dataset. Nodes represent individual genomes and are coloured according to cgST (a) or country of origin (b). Red numbers on edges indicate allelic distances between nodes. The analysis demonstrates heterogeneous population structures, including interconnected clusters enriched in Mexican ST1/RT027-associated isolates together with related Latin American genomes.

To further contextualize the Mexican isolates within the Latin American genomic landscape, additional MST analyses were performed using the Latin American genomes already included within the global dataset. Clade 2 strains formed compact ST1/RT027-associated clusters enriched in Mexican isolates and closely related genomes from other Latin American countries, supporting regional circulation and persistence of epidemic multidrug-resistant lineages. In contrast, clades 1 and 4 exhibited markedly higher allelic diversity, characterized by limited geographic clustering and predominance of unique cgSTs among isolates (Fig. 3).

### Pangenome analysis of *C. difficile*

**Fig. 4.**
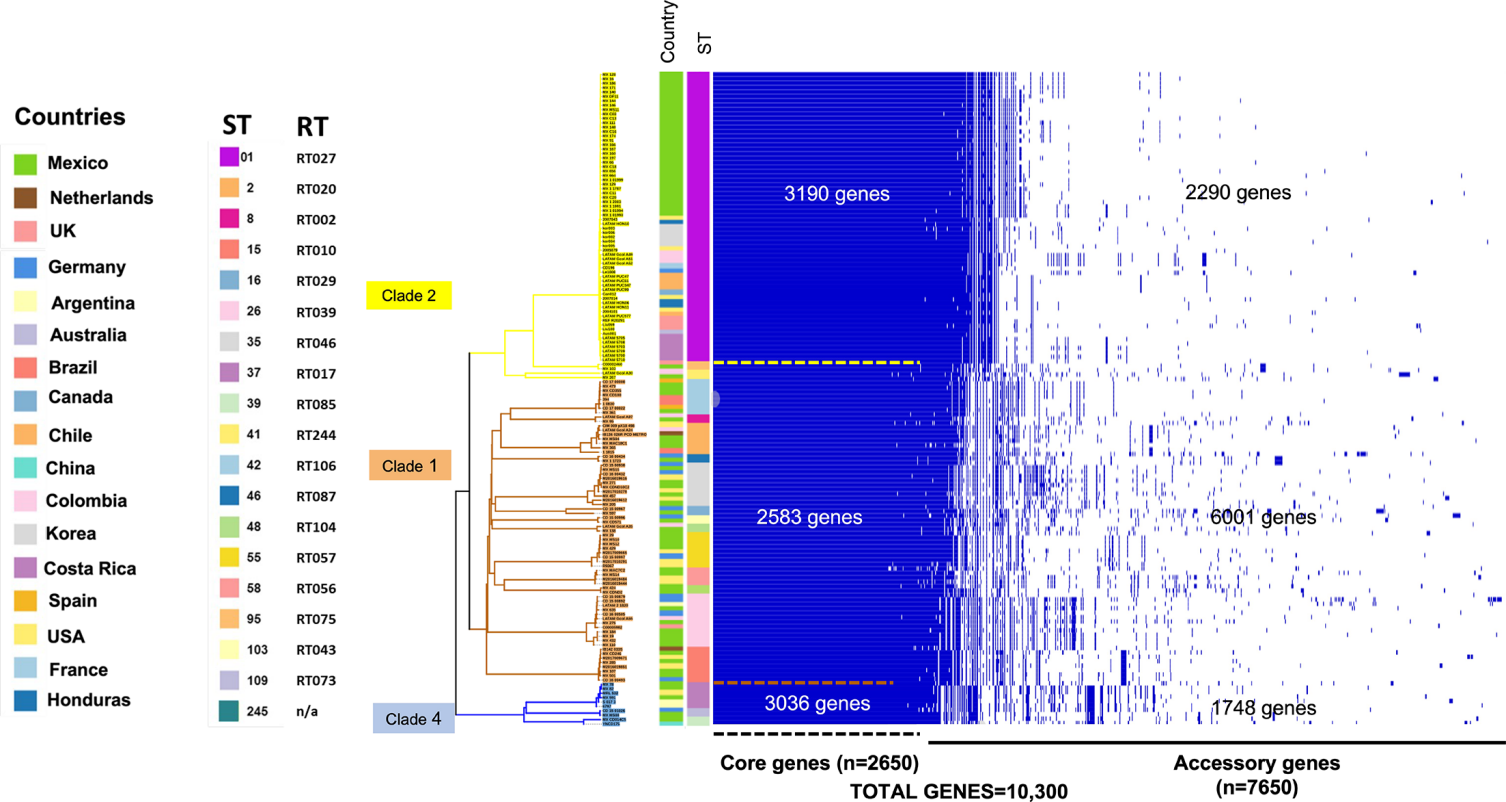
Pangenome analysis with presence/absence matrix of genes in the *C. difficile* strains. Each blue square represents a specific gene. The position of the square along the horizontal axis indicates the gene’s location in the genome. If the same gene is present in multiple genomes, it appears as a line of blue squares across several rows, each representing a different genome. The bottom dashed line represents the core genome associated within each clade, while the continuous line represents the accessory genes. This matrix was generated using Roary v3.13, phylogenetically linked to the tree created with cgMLSTfinder and visualized in Phandango.

Pangenome analysis of 151 *C*. *difficile* genomes revealed substantial differences in genetic composition among the 3 major phylogenetic clades. Clade 2, primarily composed of ST01 strains associated with epidemic lineages, contained a total of 5,480 genes, with 3,190 core genes (58%) and 2,290 accessory genes (42%). In contrast, clade 1 exhibited the highest genetic diversity, with 8,584 total genes, including 2,583 core genes (30%) and 6,001 accessory genes (70%). Clade 4 displayed a more intermediate profile with 4,784 total genes, 3,036 of which were core (63%) and 1,748 accessory (37%). These differences reflect the distinct evolutionary strategies of each clade: while clade 2 appears to have undergone recent clonal expansion (particularly among Mexican ST01 strains), clade 1 demonstrates a more dynamic, open pangenome, likely shaped by horizontal gene transfer (HGT). The pangenomic tree (Fig. 4) further supports these findings, revealing tight clustering of clade 2 strains and broader dispersion among clade 1 isolates. Overall, these results suggest that clade 2 maintains a more stable genomic structure, whereas clade 1 may have enhanced adaptability to selective pressures such as antimicrobials or host immunity.

### Comparison of virulence factors between Mexican and global strains

**Fig. 5.**
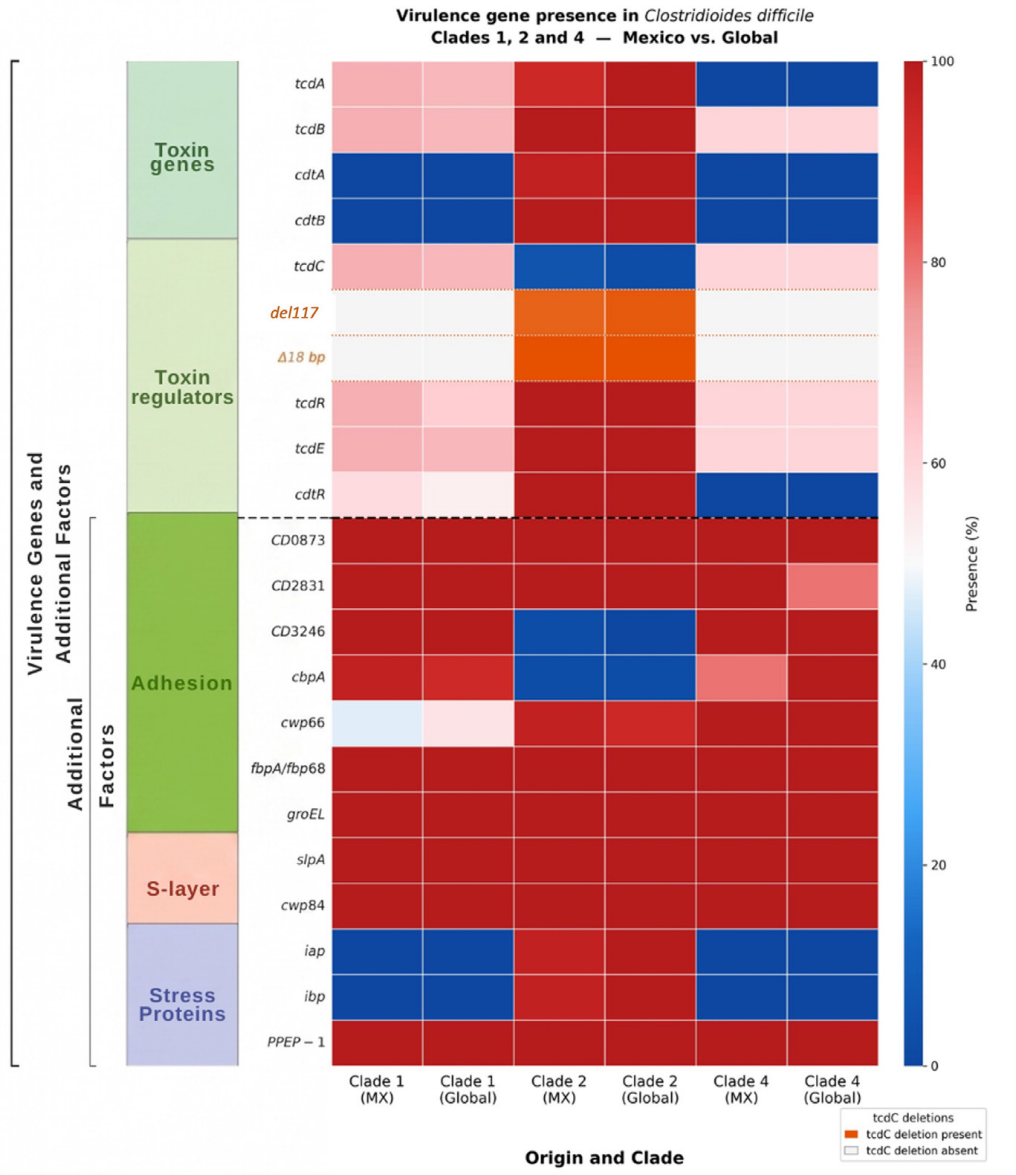
Presence of virulence genes across *C. difficile* clades 1, 2 and 4 from Mexico and global isolates. Each cell represents the percentage of isolates within each clade–origin group carrying the indicated gene, based on sequence identity thresholds from WGS data. Genes are organized into functional categories: toxin genes (*tcdA*, *tcdB*, *cdtA* and *cdtB*), toxin regulators (*tcdC*, *tcdR*, *tcdE* and *cdtR*), adhesion factors (*CD0873*, *CD2831*, *CD3246*, *cbpA*, *cwp66*, *fbpA/fbp68* and *groEL*), surface-layer proteins (*slpA* and *cwp84*) and stress proteins (*iap*, *ibp* and *PPEP-1*). Sub-rows beneath *tcdC* indicate the prevalence of the *del117* bp and Δ18 bp deletions, encoded as present (orange) or absent (light grey), independently of the main presence scale. The dashed horizontal line separates toxin-associated genes from additional virulence factors. The colour scale (blue–white–red) represents presence ranging from 0% to 100%. MX: Mexico.

To assess lineage-specific virulence factors, we compared the distribution of 20 known virulence-associated genes in 151 *C*. *difficile* genomes from Mexico and global sources (Fig. 5). As expected, clade 2 strains (mostly ST01) showed high conservation of the major toxin genes (*tcdA* and *tcdB*) and binary toxin genes (*cdtA*, *cdtB* and *cdtR*), with ≥94% prevalence in both Mexican and global isolates. This supports the hypervirulent profile of clade 2 and aligns with its known epidemiological role in severe CDI outbreaks.

Analysis of the *tcdC* regulatory gene revealed that all ST01 strains from both Mexican and global collections carried the well-characterized 18 bp deletion and the single-nucleotide deletion at position 117 (*del117*). The *del117* mutation is predicted to cause a frameshift leading to premature truncation of TcdC, a variant commonly described in epidemic RT027/ST01 strains. However, because the functional impact of *tcdC* mutations remains debated, these findings should be interpreted as lineage-associated genomic markers rather than direct evidence of increased toxin production.

In contrast, clade 4 exhibited a distinct toxigenic profile. While *tcdB* was detected in ~60% of strains, *tcdA* and binary toxin genes were absent. This pattern is consistent with the characteristic toxin gene organization previously described in several clade 4 lineages, including TcdA-negative/TcdB-positive strains associated with diverse clinical and epidemiological contexts.

**Fig. 6.**
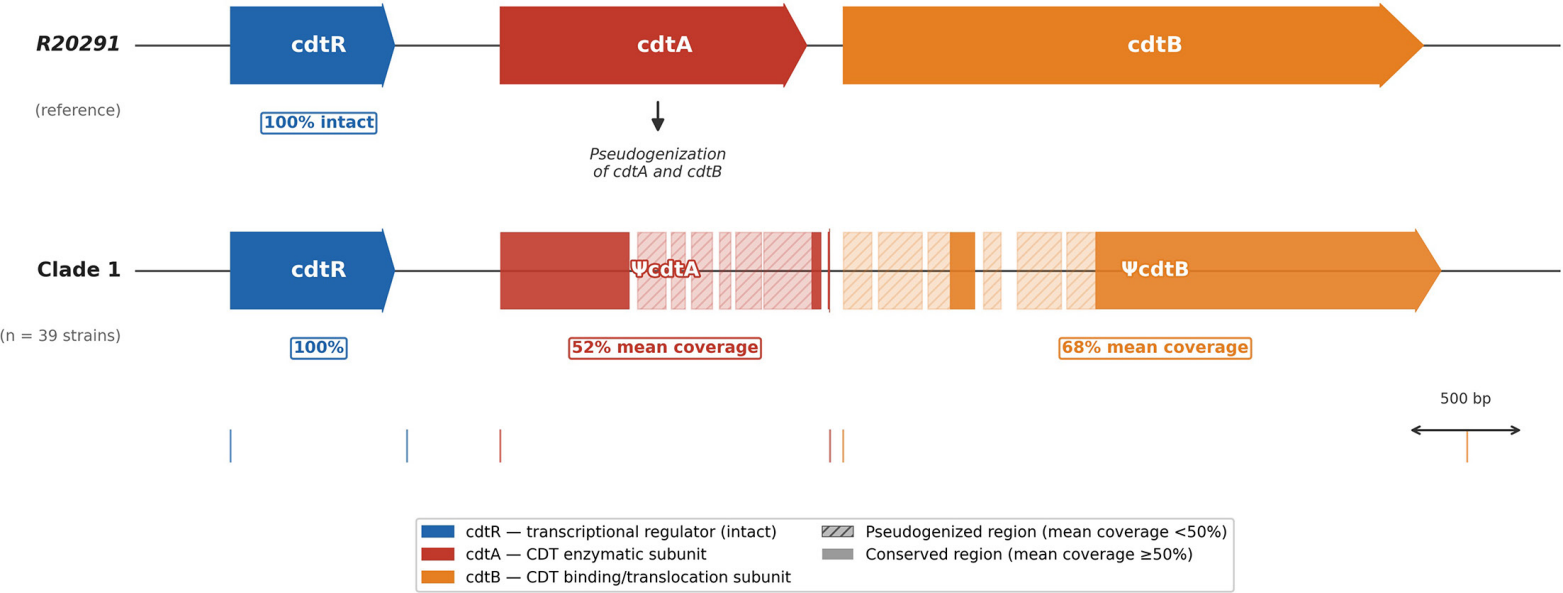
Schematic representation of the CDT locus organization in 39 clade 1 strains compared with the reference strain R20291. The regulatory gene *cdtR* remained structurally conserved and intact in all analysed strains, whereas the structural toxin genes *cdtA* and *cdtB* exhibited fragmented coverage patterns consistent with pseudogenization and partial gene degradation. Conserved regions are shown as solid blocks, while fragmented or low-coverage regions are indicated by hatched areas. Mean sequence coverage relative to the reference locus is indicated for each gene.

In clade 1, the frequency of *tcdA* and *tcdB* was similar between Mexican and global strains. Interestingly, several clade 1 isolates carried *cdtR* despite lacking detectable *cdtA* and *cdtB* genes. To determine whether this finding reflected gene misidentification or structural degradation of the CDT locus, additional comparative analyses were performed. These analyses demonstrated that clade 1 strains harboured intact and non-truncated *cdtR* open reading frames with conserved regulatory residues and preserved genomic synteny relative to the reference CDT locus from strain R20291. In contrast, *cdtA* and *cdtB* displayed fragmented sequence coverage patterns and discontinuous deletions consistent with ancestral pseudogenization of the structural toxin genes rather than complete locus absence (Figs 6 and S1). Mean sequence coverage across the locus showed complete conservation of *cdtR* (100%), whereas *cdtA* and *cdtB* exhibited partial coverage (52 and 68%, respectively), supporting the presence of a partially degraded CDT locus in these strains.

The comparison of additional virulence factors between Mexican and global strains revealed a high conservation of genes such as *fbpA/fbp68*, *groEL*, *cwp84* and *ppep-1*, across all clades and STs analysed, all of which are involved in adhesion, protein assembly and structural functions. In clade 1, genes like *CD3246* (a putative colonization factor) and *cbpA* (a collagen-binding protein) were also present in 100% of the STs, while *cwp66*, associated with adhesion, showed variability and was absent in ST2, ST8 and ST15, but present in ST16, ST35, ST46 and ST48. On the other hand, *slpA*, which encodes the main surface layer protein (S-layer), was detected only in ST35 (20%) and ST48 (100%). In clade 2, *CD3246* and *cbpA* were absent in ST01 and ST95, although *cbpA* was conserved in ST4. In clade 4, *CD3246*, *cbpA* and *cwp66* were consistently present across all STs, while *iap* and *ibp*, associated with stress response and adhesion, were absent (Table S4).

Due to the high allelic variability of *slpA*, genomes initially classified as negative by VFDB/ABRicate were reanalysed using BLASTn with relaxed identity and coverage thresholds. This secondary analysis detected *slpA* in 137 of 151 genomes (90.7%), including most clade 2 strains, with nucleotide identities ranging from 73.5% to 100% relative to the reference allele from strain 630. These findings indicate that standard virulence database thresholds substantially underestimate the prevalence of highly polymorphic loci such as *slpA*. Only 14 genomes, mainly belonging to RT046/ST35, remained negative after secondary analysis, suggesting the presence of highly divergent alleles rather than true gene absence.

Toxin B subtyping, which characterizes variants of the TcdB protein, identified 13 TcdB variants in Mexican strains and 11 variants in global isolates. The most common subtype, TcdB2.1, was detected in 45.45% of Mexican strains (35 isolates) and 45.95% of global strains (34 isolates). This subtype was primarily associated with ST01 in both groups, with 34 Mexican isolates and 33 global strains, suggesting a strong lineage association between ST01 and the TcdB2.1 variant on a global scale (Fig. S2).

### Antimicrobial resistance mechanisms in Mexican and global strains

**Fig. 7.**
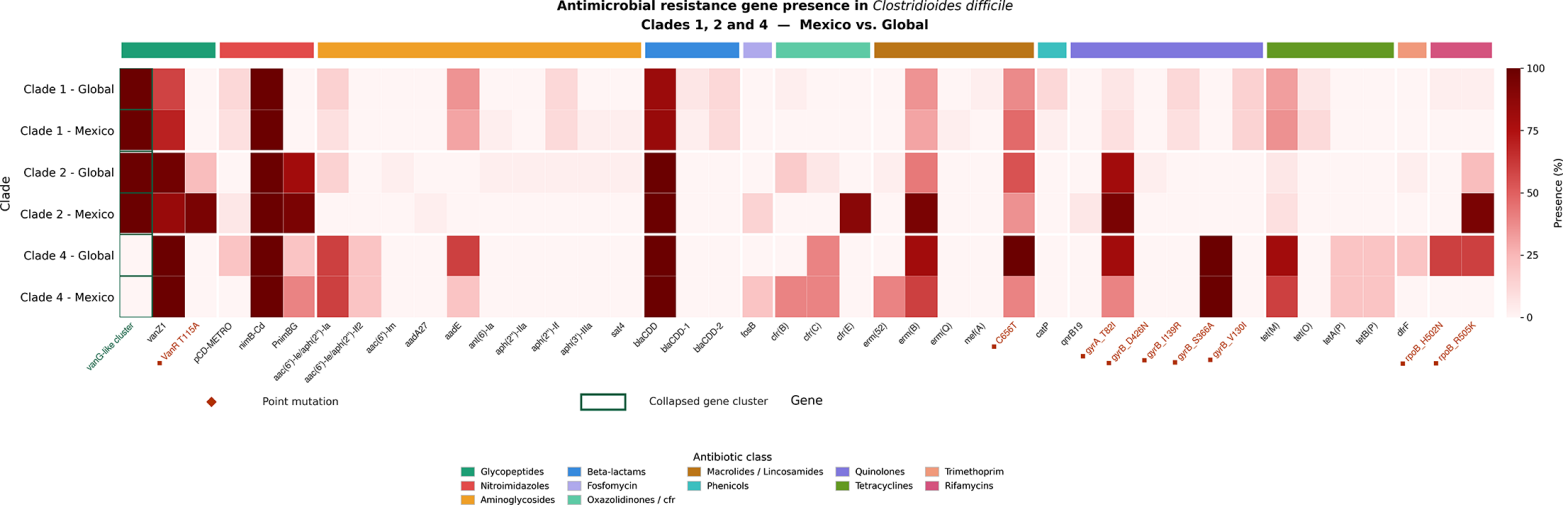
Distribution of antimicrobial resistance-associated determinants among *C. difficile* clades. Heatmap showing the prevalence (%) of antimicrobial resistance-associated genes and mutations identified among Mexican and global *C. difficile* strains grouped by phylogenetic clade. Genes are organized according to their associated antibiotic classes. The figure includes glycopeptide-associated determinants (*vanG*-like cluster and the VanR T115A substitution), nitroimidazole resistance-associated markers (*nimB*, P*nimB^G^* promoter mutation and pCD-METRO), aminoglycoside-modifying enzymes (*aac*, *aad*, *aph* and *ant*), *β*-lactam resistance genes (*blaCDD* variants), macrolide-lincosamide resistance genes (*erm*), oxazolidinone/PhLOPSA-associated *cfr* genes, quinolone resistance-associated substitutions (*gyrA*, *gyrB*), tetracycline resistance genes (*tet*) and rifamycin-associated *rpoB* mutations. Colour intensity indicates the percentage of strains carrying each determinant within each clade/geographic group.

The resistome analysis of 151 *C. difficile* strains revealed 35 antimicrobial resistance-associated determinants (AMR) and 11 point mutations associated with reduced susceptibility to various antibiotic classes. Analysis of the *vanG* cluster (*vanG*, *vanR-Cd*, *vanS-Cd *and *vanT-Cd*) revealed a similar distribution in Mexican and global strains, with near-complete prevalence in clades 1 and 2 and absence in clade 4. However, *vanZ1*, associated with vancomycin regulation, showed greater variability; in Mexican strains, it reached 69.4% (*n*=24) in clade 1 and 83.3% (*n*=29) in clade 2, whereas in global strains, it was 58.8% (*n*=19) and 97.1% (*n*=33), respectively. Additionally, the VanR T115A mutation, which may modify resistance regulation, was found in 94.4% (*n*=33) of Mexican strains, but only in 22.8% (*n*=7) of global strains from clade 2, suggesting differences in regulatory resistance mechanisms. These findings reflect a conservation of the *vanG* cluster across regions, but with variations in regulatory composition and potential expression patterns. No other vancomycin resistance-associated genes were identified in these strains (Fig. 7 and Table S5).

Multiple variants of tetracycline resistance genes were identified across the analysed genomes, including *tet(M*), *tet(O*), *tet(A/P*) and *tet(B/P*), with the highest prevalence observed in clade 4 strains. Similarly, several *β*-lactamase-associated genes were detected, including *blaCDD-1*, *blaCDD-2* and *blaCDD*, which were broadly distributed across all clades. Aminoglycoside resistance-associated determinants, particularly *aac(6')-Ie/aph(2’)-Ia*, *aadE* and *aph(3')-IIIa*, were more frequently identified among clade 4 strains, consistent with the broader accessory resistome observed in this lineage.

Regarding genetic elements associated with metronidazole resistance, only the presence of the *nimBCd* gene was universally present (100%) across all Mexican strains. Additionally, a point mutation in the promoter of the *nimB* gene (P*nimB^G^*), which is reported to enhance metronidazole resistance, was identified in all Mexican ST01 clade 2 strains (100%) and in 85% of global strains. This mutation was also detected in ST37 strains from clade 4, suggesting lineage-specific adaptation. In addition to the P*nimB^G^* promoter mutation, screening for metronidazole resistance determinants identified the presence of *nimBCd* genes and pCD-METRO-associated sequences in several isolates (Fig. 7), supporting the heterogeneous genomic basis of reduced metronidazole susceptibility among the analysed strains.

Phenotypic susceptibility testing for metronidazole, vancomycin and clindamycin was additionally performed for the 11 newly sequenced isolates generated in this study using CLSI breakpoints (Table S8). Although the P*nimB^G^* promoter mutation was detected predominantly among ST01/clade 2 strains from both Mexican and global collections, the Mexican ST01 isolates remained susceptible to metronidazole according to CLSI criteria. In contrast, elevated metronidazole MICs were observed in several non-ST01 isolates, including ST35, ST245, ST58 and ST39 strains. Similarly, most strains carrying the *vanG* operon and the VanR T115A substitution remained susceptible to vancomycin, while only one isolate (MX_MAC7C2; ST58/clade 1) exhibited high-level resistance (MIC ≥64 µg ml^−1^).

Regarding the presence of genes related to fluoroquinolone and rifampin resistance, the *gyrA_T82I* mutation, which encodes the A subunit of DNA gyrase, was identified in 94% of Mexican clade 2 strains, but only 8.3% in Mexican clade 1, and 60% of global strains from both clades. The *rpoB_R505K* mutation, linked to rifampin resistance, was detected in 94% of Mexican clade 2 strains but only in 23% of global isolates of the same clade. The *erm(B*) gene, which confers resistance to macrolides and lincosamides, was detected in 94% of Mexican clade 2 strains and 30% of Mexican clade 1 strains, but in only 42% of global isolates. The C656T mutation was 50.0% globally, but 100% within global clade 4, suggesting the existence of distinct evolutionary trajectories for macrolide resistance mechanisms in different populations.

## Discussion

In this study, we employed cgMLST to characterize the genetic diversity of *C. difficile* strains isolated in Mexico and compare them with strains from other regions. A total of 151 genomes were analysed, including 11 newly sequenced strains and 140 publicly available genomes, allowing for a detailed phylogenetic assessment of circulating strains in the country. Our study reveals previously uncharacterized patterns of genomic diversity, virulence and antimicrobial resistance-associated genes among *C. difficile* strains circulating in Mexico, highlighting regional differences that carry important global implications.

Our phylogenetic analysis demonstrated that Mexican isolates were grouped mainly into clades 1, 2 and 4, with clade 2 (ST01) strains dominating the collection. This distribution aligns with previous studies in Latin America, where the expansion of ST01 has been documented in Costa Rica, Honduras and Chile [5]. Also, this mirrors the global epidemiology where clade 2, particularly ST01/RT027 strains, has been associated with severe clinical outcomes, increased toxin production and high levels of antimicrobial resistance [3, 4]. However, our study uncovered distinct genetic traits in Mexican strains that suggest local evolutionary pressures shaping resistance patterns differently from those described in North America and Europe.

The cgMLST-based phylogenetic analysis revealed marked differences in allelic profile distribution (cgST) across the major *C. difficile* clades. Within clade 2, Mexican strains formed a clearly defined genomic cluster, comparable to the clustering previously reported for strains from Brazil and China, suggesting greater genomic conservation and lower diversification within this lineage. Notably, 40% of Mexican clade 2 isolates (14 out of 35) shared at least 1 cgST with another Mexican strain, a proportion significantly higher than that observed in clades 1 and 4 (18% and 14%, respectively). A particularly striking observation was that 5% of Mexican strains (7 out of 35) shared cgST 20031, all of which originated from Mexico City, in agreement with previous findings by Aguilar-Zamora *et al.* [8]. Although the absolute percentage is modest, the higher frequency of shared cgSTs in clade 2 supports the notion of reduced genetic variability and a more homogeneous population structure within this clade.

In contrast to clade 2, clades 1 and 4 exhibited markedly higher allelic diversity, characterized by the predominance of unique cgST profiles and the absence of clear geographic clustering among isolates, suggesting a more heterogeneous population structure. This pattern suggests increased genomic plasticity, likely driven by frequent HGT events and recombination. Previous studies have shown that clade 1 carries a wide variety of mobile genetic elements, including conjugative transposons and plasmids [9, 48] which facilitate the acquisition of antimicrobial resistance-associated genes and virulence determinants. These findings point to a potential evolutionary trade-off: while clade 2 maintains a relatively stable, epidemic-prone genomic backbone, clades 1 and 4 may be more dynamic and adaptable to fluctuating environmental and therapeutic pressures due to their greater genetic flexibility.

The regional analyses based on Latin American genomes included within the global dataset further highlighted contrasting population structures between phylogenetic clades. While clade 2 strains formed more compact ST1/RT027-associated clusters enriched in multidrug resistance-associated determinants, clades 1 and 4 exhibited substantially greater allelic diversity and limited geographic clustering. Although genomic surveillance data from Latin America remain limited, these findings support the regional circulation of epidemic clade 2 lineages and emphasize the importance of continued genomic monitoring of CDI in the region.

Pangenome analysis of 151 *C*. *difficile* genomes further highlighted these phylogenetic contrasts. Clade 2, dominated by ST01 epidemic strains, displayed the highest level of genomic conservation, with 58% of genes forming part of the core genome. This aligns with previous reports describing this lineage as clonal and genetically stable [5, 33]. Conversely, clade 1 exhibited the most pronounced genetic heterogeneity, characterized by a large proportion of accessory genes (70%), indicative of frequent acquisition of foreign genetic material and a higher propensity for recombination, consistent with the findings of Peng *et al*. [48]. Clade 4, while less studied, showed a moderately conserved genomic profile, with 63% core genes, resembling the structure described in Asian isolates [9].

The absence of clade 5 strains in the analysed collection likely reflects the limited availability of publicly sequenced Mexican genomes rather than the confirmed absence of these lineages in the region. Clade 5 strains, including RT078-associated lineages, are more frequently linked to zoonotic reservoirs and community-associated CDI in Europe and North America but remain underreported in Latin America.

### Virulence factor distribution and pathogenic potential

The virulome analysis of *C. difficile* strains revealed 20 genes associated with virulence-related functions. Subtyping of *tcdB* identified four toxin variants (B1, B2, B3 and B5), with the TcdB2.1 subtype being predominant in clade 2 ST01 strains. This is consistent with findings by Mansfield *et al*. [14], who reported TcdB2 as a hallmark of hypervirulent strains linked to severe clinical outcomes. However, in contrast to Shen *et al*. [13], who proposed horizontal transfer of *tcdB* across clades and regions, our data showed no shared subtypes between clades, supporting a model of clade-restricted evolution of this virulence determinant. Adherence and colonization-associated genes were broadly conserved across clades, with 98% of all strains carrying *CD0873*, *CD2831*, *fbpA/fbp68*, *groEL*, *cwp84* and *ppep-1*, reinforcing their role in basic survival and persistence mechanisms [49, 50]. However, it is important to note that some of these genes, such as *groEL*, a chaperonin protein involved in stress response, may contribute more to environmental survival than to virulence per se. Their high conservation may reflect their essential role in bacterial fitness rather than direct pathogenic potential. The metalloprotease previously referred to as ZmpI is currently designated PPEP-1 (pro-pro endopeptidase 1) and has been implicated in modulation of adhesion through cleavage of surface-associated proteins[51].

In contrast, several adhesion- and surface-associated genes, including CD3246, *cbpA* and highly divergent *slpA* alleles, showed clade-specific distribution patterns, being more prevalent in clades 1 and 4 than in clade 2 strains. Similar lineage-associated variation in surface proteins and adhesion-related loci has been previously described in *C. difficile* populations [52, 53]. The *slpA* gene encodes the precursor of the S-layer protein, a surface structure considered highly conserved in *C. difficile* and involved in host interaction, adherence and immune evasion [53]. After secondary BLASTn analysis using relaxed thresholds, *slpA* was detected in 90.7% of the analysed genomes, supporting its classification as part of the core genome. The wide range of nucleotide identities observed (73.5–100%) is consistent with the extensive allelic diversity previously described for this locus. Importantly, our results demonstrate that standard virulence database thresholds substantially underestimate *slpA* prevalence due to its high sequence variability. The absence of detectable hits in RT046/ST35 strains likely reflects highly divergent alleles rather than true gene loss, highlighting the need for cautious interpretation of gene presence/absence analyses for polymorphic loci.

Regarding toxin regulation, analysis of the *tcdC* gene revealed that all ST01 strains from both Mexican and global collections carried the well-characterized 18 bp deletion together with the single-nucleotide deletion at position 117 (*del117*). The *del117* mutation is predicted to introduce a frameshift resulting in premature truncation of the TcdC protein, a genetic feature commonly associated with RT027/ST01 epidemic lineages [12]. Previous studies have proposed that these alterations may contribute to altered toxin regulation and increased toxin production [54]. However, the contribution of *tcdC* mutations to hypervirulence remains controversial, as several studies have reported no direct correlation between *tcdC* truncations and toxin production levels [52]. Therefore, toxin overexpression cannot be inferred solely from genomic data without direct phenotypic or expression analyses. In this study, these *tcdC* alterations were interpreted primarily as lineage-associated genomic markers characteristic of ST01/RT027 strains.

Interestingly, several clade 1 strains carried *cdtR* despite lacking detectable *cdtA/cdtB* genes. Additional comparative analyses demonstrated that these strains harbour a partially degraded CDT locus characterized by complete conservation of *cdtR* together with extensive fragmentation of the structural toxin genes. This pattern is consistent with an ancestral pseudogenization process affecting the CDT structural components while retaining an evolutionarily conserved *cdtR* regulator. Importantly, previous experimental studies demonstrated that CdtR regulates TcdA/TcdB production primarily in RT027-associated clade 2 strains, whereas this effect has not been demonstrated in clade 1 backgrounds [17]. Therefore, the biological significance of retained *cdtR* in clade 1 strains remains unresolved and should not be interpreted as direct evidence of altered toxin production without functional validation.

Taken together, these results provide compelling evidence that *C. difficile* virulence is not only shaped by the presence or absence of key genes, but also by how these genes are arranged, regulated and expressed within distinct clades. These genomic differences may influence colonization, persistence and toxin-associated pathogenic potential among distinct lineages. The genomic characteristics of ST01/clade 2 strains are consistent with previously described epidemic RT027-associated lineages linked to increased transmission and multidrug resistance. On the other hand, the greater genomic plasticity observed in clades 1 and 4, reflected in more diverse adherence factors, may complicate long-term colonization or recurrence management.

Although non-toxigenic *C. difficile* strains are not directly associated with toxin-mediated disease, their inclusion in this study provides valuable insight into the genomic diversity and population structure of *C. difficile* circulating in Mexico. Increasing evidence suggests that non-toxigenic strains may contribute to colonization resistance by competing with toxigenic lineages and could potentially serve as biotherapeutic agents for the prevention of recurrent CDI. Furthermore, the presence of these strains may reflect ongoing evolutionary diversification within local *C. difficile* populations. Therefore, inclusion of non-toxigenic isolates contributes to a more comprehensive understanding of the epidemiology and genomic landscape of *C. difficile* in the region.

### Antimicrobial resistance-associated gene profiles and implications for treatment

A notable finding was the high frequency of the VanRT115A substitution among Mexican ST01 strains compared to global isolates. Previous studies have suggested that alterations in the *vanG* operon regulatory system may contribute to increased vancomycin MICs in certain *C. difficile* lineages [21]. However, comparison with phenotypic susceptibility data demonstrated that ST01 isolates carrying both the *vanG* operon and the VanRT115A substitution remained susceptible to vancomycin according to CLSI breakpoints, including previously reported Mexican ST01 isolates from Aguilar-Zamora *et al*. [8]. Interestingly, the only vancomycin-resistant isolate identified among the newly sequenced strains in this study (MX_MAC7C2; MIC≥64 µg ml^−1^) belonged to ST58/clade 1 rather than ST01. These observations suggest that the VanRT115A substitution alone is insufficient to explain phenotypic resistance and that additional regulatory, genomic or environmental factors likely contribute to vancomycin resistance expression in *C. difficile*.

Our resistome analysis revealed that the P*nimB^G^* promoter mutation, previously associated with reduced metronidazole susceptibility, was present in 100% of Mexican ST01 strains compared to 85% of global ST01 strains. This finding is consistent with recent reports linking *nimB* regulatory mutations to elevated metronidazole MICs in epidemic *C. difficile* lineages [23]. The high prevalence of this mutation may reflect selective pressures associated with the widespread empirical use of metronidazole in Mexico [25]. However, despite the presence of the P*nimB^G^* mutation, previous phenotypic analyses of Mexican ST01 strains by Aguilar-Zamora *et al*. [8] did not report metronidazole resistance according to CLSI breakpoints. Similarly, the ST01 isolates analysed in the present study remained susceptible based on CLSI interpretive criteria. These observations suggest that the P*nimB^G^* mutation alone is insufficient to fully predict phenotypic resistance and may instead be associated with elevated MICs or reduced susceptibility.

Metronidazole resistance in *C. difficile* is increasingly recognized as a multifactorial phenotype involving chromosomal, regulatory, metabolic and plasmid-associated mechanisms. In addition to the P*nimB^G^* promoter mutation, we identified *nimBCd* genes and pCD-METRO-associated sequences in several isolates, supporting the coexistence of multiple resistance-associated determinants among Mexican strains. Furthermore, previous studies have demonstrated that metronidazole resistance may be medium-dependent and haem-associated, with MIC values varying substantially depending on testing conditions, including haemin supplementation and light exposure [24]. Together, these findings highlight the complexity of correlating genomic resistance determinants with phenotypic susceptibility in *C. difficile*.

Phenotypic susceptibility testing performed in the newly sequenced isolates showed that Mexican ST01 strains remained susceptible to metronidazole and vancomycin according to CLSI breakpoints despite carrying resistance-associated determinants such as the P*nimB^G^* promoter mutation and the VanR T115A substitution. These findings suggest that the presence of these genomic markers alone is insufficient to predict phenotypic resistance and highlight the multifactorial nature of antimicrobial resistance in *C. difficile*. In contrast, elevated metronidazole MICs and high-level vancomycin resistance were observed in several non-ST01 isolates, indicating that additional lineage-specific mechanisms may contribute to resistance expression.

Beyond metronidazole and vancomycin, Mexican ST01 strains exhibited a notable accumulation of resistance determinants associated with fluoroquinolones, macrolides, lincosamides and rifampin, including mutations in *gyrA/gyrB*, *rpoB* and the presence of *ermB*. Similar multidrug-resistant profiles have been widely reported in epidemic RT027/ST01 lineages and are considered important drivers of their successful dissemination in healthcare settings. Aguilar-Zamora *et al*. [8] demonstrated a strong genotype–phenotype correlation between *cfrE* and linezolid resistance among Mexican ST01 isolates, supporting the epidemiological relevance of this determinant in local RT027-associated lineages. The extensive use of fluoroquinolones, clindamycin and macrolides has been strongly associated with the emergence and persistence of hypervirulent *C. difficile* lineages worldwide. Therefore, the resistome profile observed among Mexican ST01 strains suggests that antimicrobial selective pressures may contribute to the persistence and dissemination of these lineages in Mexico.

## Supplementary material

10.1099/acmi.0.001132.v3 Supplementary Material 1.

10.1099/acmi.0.001132.v3 Supplementary Material 2.

## Abbreviations

CDI: *Clostridioides difficile* infection
CDT: Clostridioides difficile binary toxin
CNGBdb: China National GeneBank DataBase
HGT: horizontal gene transfer
MSTs: minimum spanning trees
SRA: Sequence Read Archive
ST01: sequence type 01
STs: sequence types
*tpi*: triosephosphate isomerase
VFDB: Virulence Factor Database
WGS: whole-genome sequencing
