# Supplementary material for: Comparative genomic analysis of Clostridioides difficile strains in Mexico: insights into virulence and resistance
Source: Access Microbiol. 2026 Jul 29;8(7):001132.v3. doi: 10.1099/acmi.0.001132.v3 (PMC13435552; doi:10.1099/acmi.0.001132.v3)
Supplement: Supplementary file 1 [file acmi-8-01132-s002.pdf]

cgMLST tree and data set

https://online.phyloviz.net/index
Name: C. difficile Mexico 2025

Supplementary Figure 1

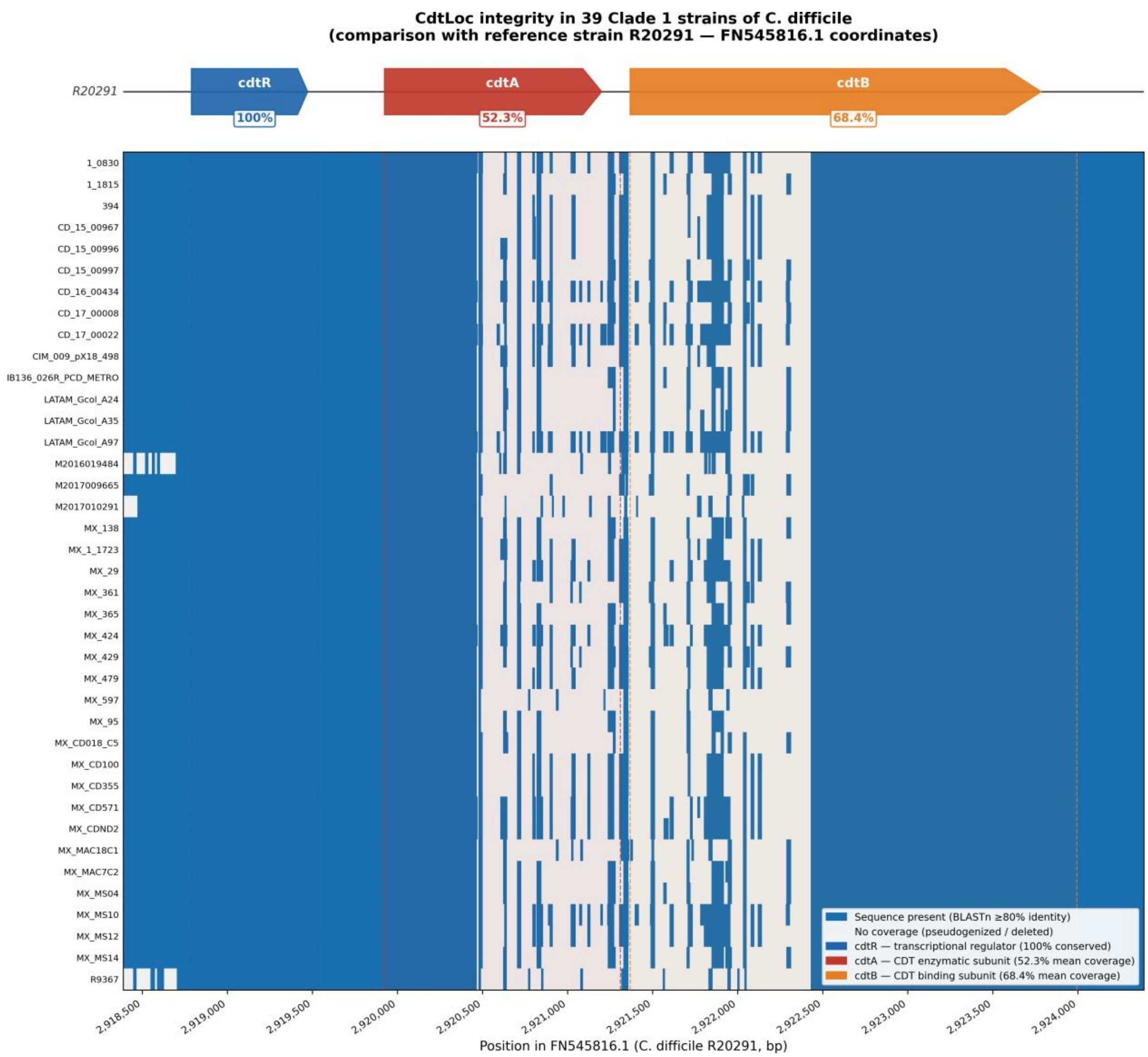

Supplementary figure 1. Per-strain nucleotide coverage of the CDT locus across 39 Clade 1 strains of *Clostridioides difficile*.

Top: CDT locus organization of reference strain R20291 drawn to scale. Bottom: heatmap of per-position nucleotide coverage for each strain against the CdtLoc reference region (FN545816.1: 2,916,789–2,925,996). Blue indicates positions covered by BLASTn alignments (≥80% identity); gray indicates no coverage. Dashed lines delimit gene boundaries. Complete conservation of cdtR contrasts with the fragmented coverage pattern of cdtA and cdtB, reflecting progressive pseudogenization of the CDT structural subunits.

Supplementary Figure 2

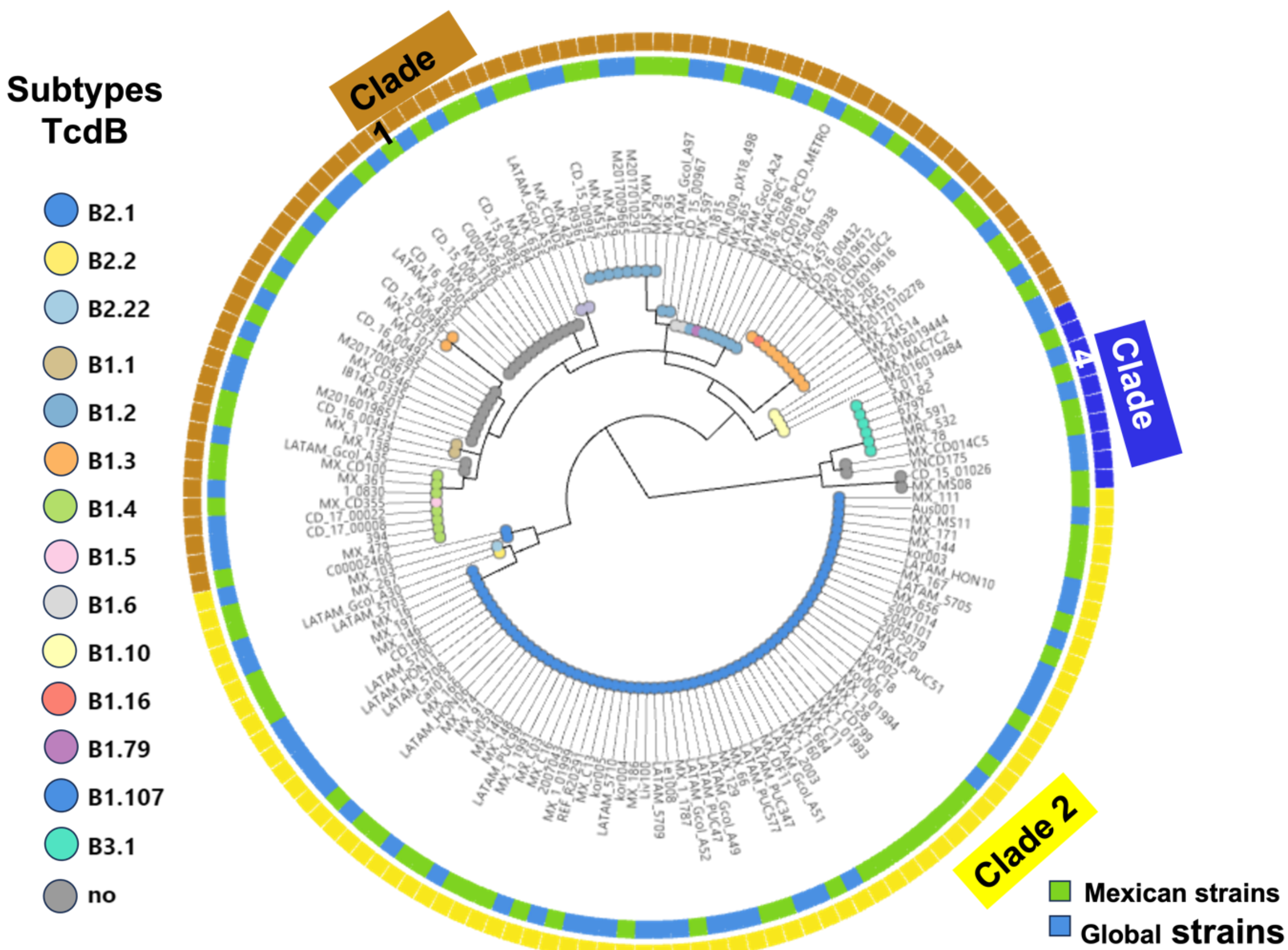

Supplementary Figure 2. *Circular phylogenetic tree of Mexican and global Clostridioides difficile strains annotated with TcdB toxin subtypes.* The inner ring represents the different *TcdB*

subtypes using a color code, while the outer ring indicates the geographic origin of the strains: Mexican (green) and global (blue). Phylogenetic clades are marked on the outer edge of the diagram (clade 1 in brown, clade 2 in yellow, and clade 4 in blue). Each label on the tree branches corresponds to an individual strain.
